# ﻿Two new species of *Dendrostoma* (Erythrogloeaceae, Diaporthales) associated with *Castaneamollissima* canker disease in China

**DOI:** 10.3897/mycokeys.108.128197

**Published:** 2024-09-13

**Authors:** Ning Jiang, Xiaojie Qi, Baoxin Qi, Fang Cai, Han Xue, Yong Li

**Affiliations:** 1 Key Laboratory of Biodiversity Conservation of National Forestry and Grassland Administration, Ecology and Nature Conservation Institute, Chinese Academy of Forestry, Beijing 100091, China Ecology and Nature Conservation Institute, Chinese Academy of Forestry Beijing China; 2 Forest Pest Control and Quarantine Station of Xining City, Qinghai 810099, Xining, China Forest Pest Control and Quarantine Station of Xining City Xining China

**Keywords:** Ascomycota, Chinese chestnut, molecular phylogeny, plant disease, Sordariomycetes, taxonomy

## Abstract

The genus *Dendrostoma* is known to inhabit tree barks associated with branch canker diseases in China and several countries of Europe. Previous studies indicated that species of *Dendrostoma* prefer inhabiting fagaceous hosts, especially species of *Castanea*. In the present study, we obtained four isolates from cankered branches of Chinese chestnut (*C.mollissima*) in Rizhao City, Shandong Province, China. Morphological comparisons and phylogenetical analyses of a combined ITS-*tef1*-*rpb2* sequence matrix were conducted, which revealed two new species named *Dendrostomarizhaoense***sp. nov**. and *D.tianii***sp. nov.** The new taxa are compared with other *Dendrostoma* species and comprehensive descriptions and illustrations are provided herein.

## ﻿Introduction

The genus *Dendrostoma* (Erythrogloeaceae, Diaporthales) was proposed by Fan et al. (2018) with *D.mali* from *Malusspectabilis* (Rosaceae) as the type species. Meanwhile, *D.osmanthi* from *Osmanthusfragrans* (Oleaceae) and *D.quercinum* from *Quercusacutissima* (Fagaceae) in China were introduced (Fan et al. 2018). Subsequently, an old species *Amphiportheleiphaemia* was transferred to this genus as *D.leiphaemia*, which inhabited *Quercus* spp. in Europe (Senanayake et al. 2018).

Jiang et al. (2019) studied samples collected from *Castaneamollissima* and *Quercus* spp. (Fagaceae) based on both morphological and molecular evidence, introducing 10 additional species named *D.aurorae*, *D.castaneae*, *D.castaneicola*, *D.chinense*, *D.dispersum*, *D.parasiticum*, *D.qinlingense*, *D.quercus*, *D.shaanxiense* and *D.shandongense*. Subsequently, European species of *Dendrostoma* were studied, with 3 new species and a new combination were described, viz. *D.atlanticum* and *D.castaneum* from *Castaneasativa*, *D.creticum* from *Quercuscoccifera* and *D.istriacum* from *Q.ilex* (Jaklitsch and Voglmayr 2019). Later, *D.donglingense* was discovered from *Quercusmongolica* in China (Zhu et al. 2019); *D.luteum* was proposed based on the collection from *Castaneasativa* in England (Crous et al. 2020); *D.covidicola* was introduced from *Fagussylvatica* (Fagaceae) in China (Samarakoon et al. 2021); *D.elaeocarpi* was introduced from *Elaeocarpusdecipiens* (Elaeocarpaceae) in China (Chen et al. 2022). Before the present study, 22 species of *Dendrostoma* were accepted. Of these, 16 species were discovered in China, and the rests in Austria, Croatia, England, France, Greece, Italy, Netherlands and Spain in Europe. Additionally, 19 species of this genus were found on the tree barks of Fagaceae, and the other three species on Elaeocarpaceae, Oleaceae and Rosaceae, respectively.

Morphologically, *Dendrostoma* is characterised by having multiguttulate and bicellular ascospores that are constricted at the septum and acervular or pycnidial conidiomata, with subcylindrical to ampulliform conidiogenous cells and hyaline to olivaceous, aseptate conidia (Fan et al. 2018; Jaklitsch and Voglmayr 2019; Jiang et al. 2019). However, several species share same hosts and similar sexual and asexual characters. For example, *D.chinense* and *D.shandongense* both occurred on branches and twigs of *Castaneamollissima* with similar conidial shape and size (Jiang et al. 2019). Hence, sequence data are necessary during species identification and distinguishment (Chen et al. 2022).

Additional sample collections of *Dendrostoma* were conducted in consideration of rich species diversity of this genus on the host *Castaneamollissima* in China. In this study, we collected diseased branches of Chinese chestnut and obtained fungal isolates. Species identification was conducted following the approaches described in Chen et al. (2022).

## ﻿Materials and methods

### ﻿Sample collection, morphology and isolation

In 2022 and 2023, investigations to collect *Dendrostoma* samples were conducted in Shandong Province, China. Cankered branches with or without fungal fruiting bodies were collected and packed in paper bags. Then samples were returned to observed for fungal isolation in three days.

Diseased branches with fruiting bodies were isolated by removing spore masses from ascomata or conidiomata on clean PDA (PDA, 200 g potatoes, 20 g dextrose, 20 g agar per L) plates and incubating at 25 °C until spores germinated. Single germinated spores were further transferred to the new PDA plates and incubated at 25 °C in the dark. Diseased branches without fruiting bodies were isolated by the following steps. Firstly, discolored barks were surface- sterilized for 5 min in 75% ethanol, rinsed for 1 min in distilled water and blotted on dry sterile filter paper. Secondly, diseased tissues were cut into 0.5 cm × 0.5 cm pieces using a double-edge blade, and transferred on the surface of PDA, which were incubated at 25 °C to obtain cultures. Thirdly, hyphal tips of the cultures growing from the diseased tissues were transferred to new PDA plates under a dissecting stereomicroscope using sterile needles. The cultures were deposited in China Forestry Culture Collection Center (CFCC, http://cfcc.caf.ac.cn/; accessed), and the specimens in the herbarium of the Chinese Academy of Forestry (CAF, http://museum.caf.ac.cn/).

Observation and description of new *Dendrostoma* species was based on fruiting bodies naturally formed on the host barks and PDA plates. Ascostromata and conidiomata were hand sectioned using a double-edged blade under a dissecting microscope. At least 10 conidiomata/ascostromata, 10 asci and 50 conidia/ascospores were measured to calculate the mean size and standard deviation. Measurements are reported as maximum and minimum in parentheses and the range representing the mean plus and minus the standard deviation and the number of measurements is given in parentheses. Microscopy photographs were captured with a Nikon Eclipse 80i compound microscope equipped with a Nikon digital sight DS-Ri2 high definition colour camera, using differential interference contrast illumination.

### ﻿DNA extraction, PCR amplification, and sequencing

The total DNA was obtained from fresh mycelia growing on PDA following Doyle and Doyle (1990). Three loci including the internal transcribed spacer region rDNA (ITS), translation elongation factor 1-alpha (*tef1*) and RNA polymerase II second largest subunit (*rpb2*) were amplified using primers and conditions listed in Table 1. The Polymerase chain reactions (PCR) products were assayed via electrophoresis in 2% agarose gels. DNA sequencing was performed using an ABI PRISM 3730XL DNA Analyser with a BigDye Terminator Kit v.3.1 (Invitrogen, Waltham, MA, USA) at the Shanghai Invitrogen Biological Technology Company Limited (Beijing, China).

**Table 1.** Primers and PCR protocols.

Gene Regions	Primers	PCR conditions	References
ITS	ITS5/ITS4	95 °C for 4 min, 35 cycles of 94 °C for 45 s, 48 °C for 1 min, and 72 °C for 2 min, 72 °C for 10 min	White et al. (1990)
*rpb2*	fRPB2-5f/fRPB2-7cR	95 °C for 5 min, 35 cycles of 95 °C for 1 min, 55 °C for 75 s, and 72 °C for 2 min, 72 °C for 10 min	Liu et al. (1999)
*tef1*	728F/986R	94 °C for 3 min, 35 cycles of 94 °C for 30 s, 54 °C for 50 s, and 72 °C for 2 min, 72 °C for 10 min	Carbone and Kohn (1999)

### ﻿Sequence alignment and Phylogenetic analyses

The obtained sequences of ITS, *tef1* and *rpb2* were assembled using SeqMan software version 7.1.0 (DNASTAR Inc., WI) and subjected to BLASTn search against the GenBank nucleotide database at National Center for Biotechnology Information (NCBI) to identify closely related sequences. Sequences data of related taxa were obtained from previous publications (Fan et al. 2018; Jaklitsch and Voglmayr 2019; Jiang et al. 2019; Zhu et al. 2019; Crous et al. 2020; Samarakoon et al. 2021; Chen et al. 2022) and downloaded from the GenBank database (Table 2). The sequences were aligned using MAFFT v.7 online web server (http://mafft.cbrc.jp/alignment/server/index.html, Katoh et al. 2019) under default settings. The maximum likelihood (ML) phylogenic analysis was run in the CIPRES Science Gateway platform (Miller et al. 2010), using RAxMLHPC2 on the XSEDE (v. 8.2.10) tool under the GTR substitution model and 1000 non-parametric bootstrap replicates. Bayesian analysis was performed using MrBayes v. 3.2.6 on XSEDE at the CIPRES with four simultaneous Markov Chain runs for 1000000 generations. The resulting trees were visualised in FigTree v. 1.4.1 (Rambaut 2012).

**Table 2.** GenBank accession numbers of *Dendrostoma* species.

Species	Isolates	GenBank accession numbers			References
		ITS	*tef1*	*rpb2*	
*Dendrostomaatlanticum*	CBS 145804*	MN447223	MN432167	MN432160	Jaklitsch and Voglmayr (2019)
*Dendrostomaaurorae*	CFCC 52753*	MH542498	MH545447	MH545405	Jiang et al. (2019)
*Dendrostomaaurorae*	CFCC 52754	MH542499	MH545448	MH545406	Jiang et al. (2019)
*Dendrostomacastaneae*	CFCC 52745*	MH542488	MH545437	MH545395	Jiang et al. (2019)
*Dendrostomacastaneae*	CFCC 52746	MH542489	MH545438	MH545396	Jiang et al. (2019)
*Dendrostomacastaneicola*	CFCC 52743*	MH542496	MH545445	MH545403	Jiang et al. (2019)
*Dendrostomacastaneicola*	CFCC 52744	MH542497	MH545446	MH545404	Jiang et al. (2019)
*Dendrostomacastaneum*	CBS 145803*	MN447225	MN432169	MN432162	Jaklitsch and Voglmayr (2019)
*Dendrostomachinense*	CFCC 52755*	MH542500	MH545449	MH545407	Jiang et al. (2019)
*Dendrostomachinense*	CFCC 52756	MH542501	MH545450	MH545408	Jiang et al. (2019)
*Dendrostomacovidicola*	GZCC 20-0355*	MW261327	MW262894	MW262892	Samarakoon et al. (2021)
*Dendrostomacreticum*	CBS 145802*	MN447228	MN432171	MN432163	Jaklitsch and Voglmayr (2019)
*Dendrostomadispersum*	CFCC 52730	MH542467	MH545416	MH545374	Jiang et al. (2019)
*Dendrostomadispersum*	CFCC 52728*	MH542469	MH545418	MH545376	Jiang et al. (2019)
*Dendrostomadonglingense*	CFCC 53148*	MN266206	MN315480	MN315491	Zhu et al. (2019)
*Dendrostomadonglingense*	CFCC 53149	MN266207	MN315481	MN315492	Zhu et al. (2019)
*Dendrostomaelaeocarpi*	CFCC 53113*	MK432638	MK578114	MK578096	Chen et al. (2022)
*Dendrostomaelaeocarpi*	CFCC 53114	MK432639	MK578115	MK578097	Chen et al. (2022)
*Dendrostomaistriacum*	CBS 145801*	MN447229	MN432172	MN432164	Jaklitsch and Voglmayr (2019)
*Dendrostomaleiphaemia*	CFCC 54038*	MN545571	MN551288	MN551291	Chen et al. (2022)
*Dendrostomaleiphaemia*	CFCC 54039	MN545572	MN551289	MN551292	Chen et al. (2022)
*Dendrostomaleiphaemia*	CFCC 54040	MN545573	MN551290	MN551293	Chen et al. (2022)
*Dendrostomaleiphaemia*	CBS 145800	MN447230	MN432173	MN432165	Jaklitsch and Voglmayr (2019)
*Dendrostomaluteum*	IMI506898*	MN648726	MN812768	NA	Crous et al. (2020)
*Dendrostomamali*	CFCC 52102*	MG682072	MG682052	MG682032	Fan et al. (2018)
*Dendrostomaosmanthi*	CFCC 52106*	MG682073	MG682053	MG682033	Fan et al. (2018)
*Dendrostomaosmanthi*	CFCC 52108	MG682074	MG682054	MG682034	Fan et al. (2018)
*Dendrostomaparasiticum*	CFCC 52762*	MH542482	MH545431	MH545389	Jiang et al. (2019)
*Dendrostomaparasiticum*	CFCC 52764	MH542483	MH545432	MH545390	Jiang et al. (2019)
*Dendrostomaqinlingense*	CFCC 52732*	MH542471	MH545420	MH545378	Jiang et al. (2019)
*Dendrostomaqinlingense*	CFCC 52733	MH542472	MH545421	MH545379	Jiang et al. (2019)
*Dendrostomaquercinum*	CFCC 52103*	MG682077	MG682057	MG682037	Fan et al. (2018)
*Dendrostomaquercinum*	CFCC 52104	MG682078	MG682058	MG682038	Fan et al. (2018)
*Dendrostomaquercus*	CFCC 52739*	MH542476	MH545425	MH545383	Jiang et al. (2019)
*Dendrostomaquercus*	CFCC 52738	MH542477	MH545426	MH545384	Jiang et al. (2019)
***Dendrostomarizhaoense***	CFCC 57559*	**PP965514**	**PP957893**	**PP957897**	**Present study**
***Dendrostomarizhaoense***	**CFCC 57560**	**PP965515**	**PP957894**	**PP957898**	**Present study**
*Dendrostomashaanxiense*	CFCC 52741*	MH542486	MH545435	MH545393	Jiang et al. (2019)
*Dendrostomashaanxiense*	CFCC 52742	MH542487	MH545436	MH545394	Jiang et al. (2019)
*Dendrostomashandongense*	CFCC 52759*	MH542504	MH545453	MH545411	Jiang et al. (2019)
*Dendrostomashandongense*	CFCC 52760	MH542505	MH545454	MH545412	Jiang et al. (2019)
***Dendrostomatianii***	CFCC 58140*	**PP965516**	**PP957895**	**NA**	**Present study**
***Dendrostomatianii***	**CFCC 58141**	**PP965517**	**PP957896**	**NA**	**Present study**

Note. Ex-type strains are indicated with * after the collection number; “NA” indicates unavailable sequences; sequences produced in the current study are in bold.

## ﻿Results

### ﻿Phylogenetic analyses

**Figure 1.**
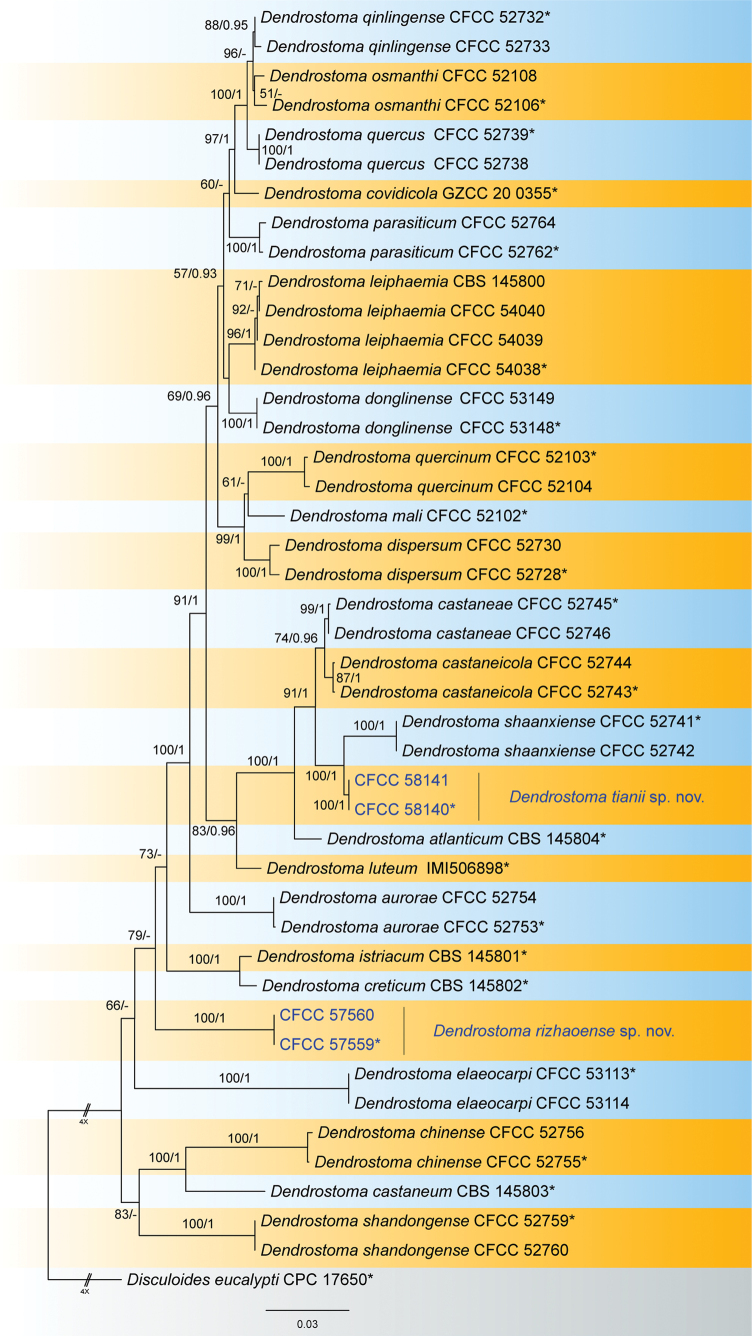
Maximum likelihood tree generated from combined ITS, *tef1* and *rpb2* sequence data. Bootstrap support values ≥ 50% and Bayesian posterior probabilities ≥ 0.90 are demonstrated at the branches. Isolates from the present study are indicated in blue, and ex-type strains are marked with *.

The combined ITS, *tef1* and *rpb2* dataset consisted of 44 strains, with *Disculoideseucalypti* (CPC 17650) as the outgroup taxon (Table 2). The final alignment comprised 2018 characters (ITS: 509, *tef1*: 434, *rpb2*: 1075), including gaps. The final ML optimisation likelihood value of the best RAxML tree was -8904.92, and the matrix had 636 distinct alignment patterns, with 10.07% undetermined characters or gaps. Estimated base frequencies were as follows: A = 0.238059, C = 0.283419, G = 0.251935, T = 0.226587; substitution rates AC = 1.976244, AG = 3.303507, AT = 1.131972, CG = 0.925964, CT = 7.189207, GT = 1.0; gamma distribution shape parameter α = 0.238059. The RAxML and Bayesian analyses yielded a similar tree topology. The topology of our phylogenetic tree is nearly identical to previous publications (Samarakoon et al. 2021; Chen et al. 2022). Isolates CFCC 57559 and CFCC 57560 formed a new clade distinct from any known species; and CFCC 58140 and CFCC 58141 formed a new clade sister to *Dendrostomashaanxiense* shown in the phylogram (Fig. 1).

### ﻿Taxonomy

#### ﻿ Dendrostoma rizhaoense

Taxon classificationFungiDiaporthalesErythrogloeaceae

Ning Jiang sp. nov.

8BAC4DB0-387C-5925-8B5B-4047D283CC23

 854073

Fig. 2

##### Etymology.

Named after the collection site of the type specimen, Rizhao City.

##### Holotype.

CAF800092.

##### Description.

***Sexual morph***: Undetermined. ***Asexual morph***: Conidiomata formed on PDA, pycnidial, ostiolated, conical to pulvinate, occurring separately, brown, 150–350 μm high, 200–450 μm diam.; wall of several layers of faint yellow textura angularis. Conidiophores reduced to conidiogenous cells. Conidiogenous cells lining the inner walls of the cavity, hyaline, smooth, subcylindrical to ampulliform, 8–27.5 × 3–5.5 μm. Conidia hyaline, aseptate, smooth, multiguttulate, thin-walled, ellipsoid to fusoid, straight, (5.6–)6.4–8.8(–10.7) × (2.4–)2.7–3.8(–4.5) μm, l/w = (1.5–)1.8–3.1(–3.8) (n = 50).

**Figure 2.**
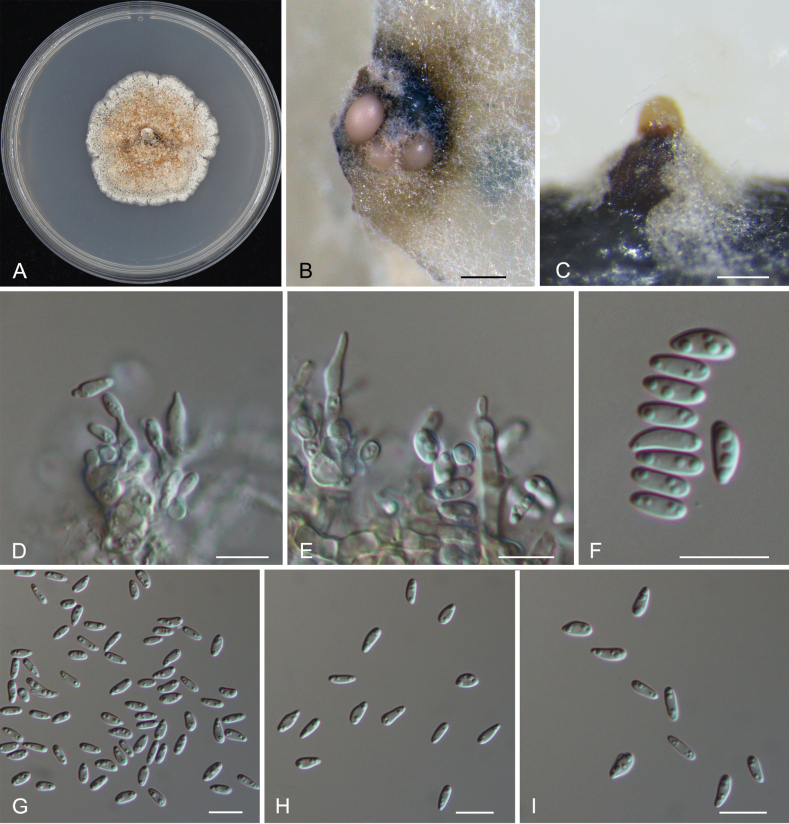
*Dendrostomarizhaoense* (CAF800092, holotype) **A** colony on the PDA plate **B, C** conidiomata formed on the PDA plate after 28 days **D, E** conidiogenous cells with attached conidia **F–I** conidia. Scale bars: 200 µm (**B**); 100 µm (**C**); 10 µm (**D–I**).

##### Culture characteristics.

Colonies on PDA flat, initially white, becoming dark orange after 2 weeks, texture uniform, producing conidiomata after 4 weeks.

##### Materials examined.

China • Shandong Province, Rizhao City, Lanshan District, Huangdun Town, on cankered barks of *Castaneamollissima*, 16 October 2022, Jiang Ning (CAF800092, ***holotype***); ex-type cultures CFCC 57559 and CFCC 57560.

##### Notes.

Two isolates of *Dendrostomarizhaoense* from *Castaneamollissima* formed a distinct clade in the phylogram of this genus based on the combined sequence of ITS, *tef1* and *rpb2* (Fig. 1). With two new species proposed in the present study, nine species of *Dendrostoma* were recorded from the host *Castaneamollissima*, viz. *D.aurorae*, *D.castaneae*, *D.castaneicola*, *D.chinense*, *D.parasiticum*, *D.rizhaoense*, *D.shaanxiense*, *D.shandongense* and *D.tianii* (Jiang et al. 2019). Morphologically, *D.rizhaoense* (6.4–8.8 μm) has shorter conidia than *D.castaneae* (10.4–12.3 μm), *D.castaneicola* (10.5–12.8 μm), *D.parasiticum* (9.3–11.7 μm), *D.shaanxiense* (9.5–11.1 μm) and *D.tianii* (9.5–11.1 μm); *D.rizhaoense* (2.7–3.8 μm) has wider conidia than *D.aurorae* (2.3–2.6 μm), but narrower conidia than *D.shandongense* (3.8–4.3 μm) (Jiang et al. 2019). In addition, *D.rizhaoense* is similar to *D.chinense* in conidial size, but differs in the phylogenetical position.

#### ﻿ Dendrostoma tianii

Taxon classificationFungiDiaporthalesErythrogloeaceae

Ning Jiang sp. nov.

7CDB78F9-ECD0-5C5E-AA77-BFB2B97462B4

 854074

Fig. 3

##### Etymology.

Named after the Chinese taxonomist Prof. Dr. Tian Chengming.

##### Holotype.

CAF800093.

##### Description.

***Sexual morph***: Pseudostromata erumpent, consisting of an inconspicuous ectostromatic disc, semi-immersed, causing a pustulate bark surface, 800–1750 µm diam. Ectostromatic disc flat or concave, brown, sometimes concealed by ostioles, surrounded by bark flaps, 350–750 µm diam.; central column yellowish to brownish. Stromatic zones lacking. Perithecia conspicuous, umber to fuscous black, 300–450 µm diam. Ostioles 4–9 per disc, flat in the disc or sometimes slightly projecting, cylindrical, covered by an orange, umber to fuscous black crust, 55–80 µm diam. Paraphyses slightly deliquescent. Asci fusoid to slightly fusiform, 8-spored, ascospores regularly disposed, with an apical ring, 55–75 × 13–16.5 µm. Ascospores hyaline, fusoid to cylindrical, smooth, straight, bicellular, (17.5–)18.7–22.1(–23.8) × (4.6–)5.4–6.8(–7) μm, l/w = (2.8–)3–3.7(–4.1) (n = 50), with a hyaline, subconical to filiform appendage 5.5–8.5 × 2–2.5 μm at each end. ***Asexual morph***: Conidiomata formed on host barks acervular, conical to pulvinate, occurring separately, pale brown, immersed to semi-immersed, 300–400 μm high, 250–350 μm diam.; wall of several layers of faint yellow textura angularis; central column beneath the disc, yellow. Conidiogenous cells lining the inner walls of the cavity, hyaline, smooth, subcylindrical, 6.5–10.5 × 2.5–5 μm. Conidia hyaline, aseptate, smooth, multiguttulate, thin-walled, ellipsoid, straight or slightly curved, (8.1–)9.5–11.1(–12.2) × (2.5–)2.6–3.2(–3.4) μm, l/w = (3–)3.2–4.1(–4.6) (n = 50).

**Figure 3.**
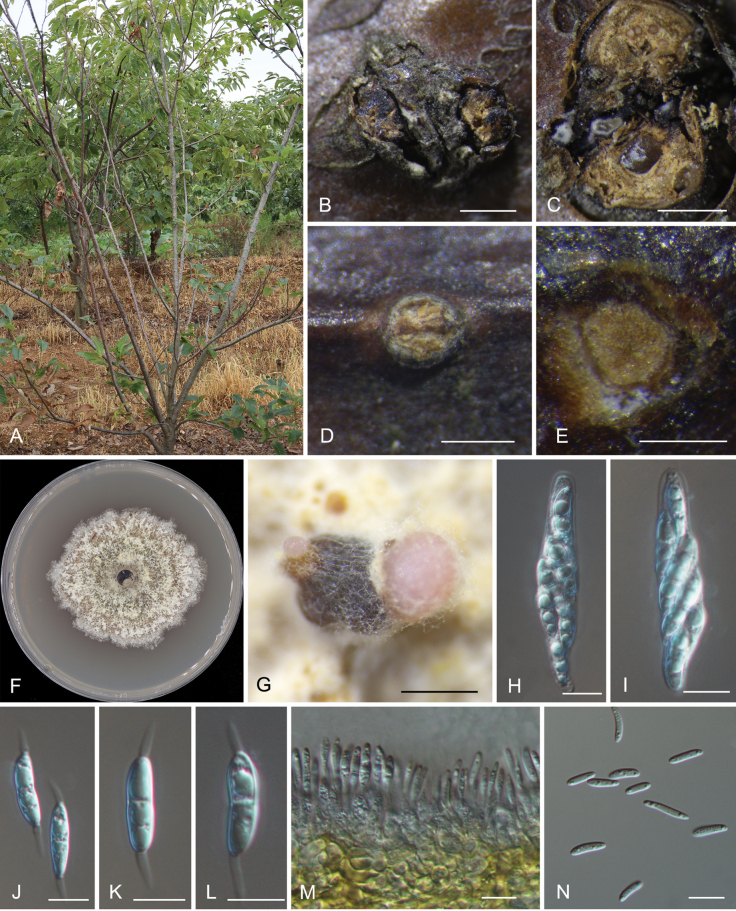
*Dendrostomatianii* (CAF800093, holotype) **A** a diseased Chinese chestnut tree **B** habit of psedostromata **C** transverse section through the pseudostroma **D** habit of a conidioma **E** transverse section through the conidioma **F** colony on the PDA plate **G** conidiomata formed on the PDA after 28 days **H, I** ascus **J–L** ascospores **M** conidiogenous cells with attached conidia **N** conidia. Scale bars: 1000 µm (**B, C**); 200 µm (**D**); 300 µm (**E**); 500 µm (**G**); 10 µm (**H–N**).

##### Culture characteristics.

Colonies on PDA flat, initially white, becoming pale brown after 2 weeks, texture uniform, producing conidiomata after 4 weeks.

##### Materials examined.

China • Shandong Province, Rizhao City, Wulian County, Songbai Town, on cankered branches of *Castaneamollissima*, 15 July 2023, Jiang Ning (CAF800093, ***holotype***); ex-type culture CFCC 58140 • Shandong Province, Rizhao City, Wulian County, Shichang Town, on cankered branches of *C.mollissima*, 15 July 2023, Jiang Ning (BL013); culture CFCC 58141.

##### Notes.

*Dendrostomatianii* is phylogenetically close to *D.shaanxiense* (Fig. 1). These two species share the same host *Castaneamollissima*, and are both distributed in China; *D.tianii* in Shandong Province, while *D.shaanxiense* in Shaanxi Province. In addition, they have similar conidia in shape and size. However, they are distinguished by sequence data (nucleotide differences in the ITS: 25/400 (6.25%), 7 insertion; in *tef1*: 9/400 (2.25%), 7 insertion) (Jiang et al. 2019).

## ﻿Discussion

*Dendrostomarizhaoense* sp. nov. and *D.tianii* sp. nov. are proposed in the present study, which increase the species number of this genus from 22 to 24 (http://www.indexfungorum.org/, accessed on 20 May 2024). All these species of *Dendrostoma* are studied in both morphology and sequence data. However, all the species are discovered in east Asia and Europe (Fan et al. 2018; Jaklitsch and Voglmayr 2019; Jiang et al. 2019; Zhu et al. 2019; Crous et al. 2020; Samarakoon et al. 2021; Chen et al. 2022), many potential new species remain to be found from tree hosts in the other areas, such as Africa, America and Australia in the future.

Species of *Dendrostoma* are potentially canker pathogens to their hosts, according to the symptoms recorded in Jiang et al. (2019) and Crous et al. (2020), as well as in this study. *D.castaneum* causes canker disease symptoms on *Castaneasativa* in artificial inoculation (Défago 1937), and is considered as a weak wound pathogen to the host (Phillips and Burdekin 1982); *Dendrostoma* sp. (as *Cryptodiaporthecastanea*) shows pathogenicity ability in Japanese chestnut (Kobayashi 1970). However, Jaklitsch and Voglmayr (2019) did not observe obvious disease symptoms exhibited by *Castanea* and *Quercus* hosts infected by species of *Dendrostoma*. Future pathogenicity tests based on Koch’s postulates are needed to be conducted to confirm pathogenicity ability of *Dendrostoma* to their hosts.

Currently, 21 species of this genus were discovered from the plant family Fagaceae, of which nine species from Chinese chestnut (Table 3), three species from European chestnut, and the other from *Fagus* and *Quercus* hosts (Fan et al. 2018; Jaklitsch and Voglmayr 2019; Jiang et al. 2019; Zhu et al. 2019; Crous et al. 2020; Samarakoon et al. 2021; Chen et al. 2022). In China, there are more than 320 Fagaceae species, which indicates rich cryptic species diversity of *Dendrostoma* to be discovered in China in the future.

**Table 3.** Morphology of *Dendrostoma* species from *Castaneamollissima*.

Species	Conidial length (μm)	Conidial width (μm)	Length/width ratio
*D.aurorae*	8.1–9.8	2.3–2.6	3.2–4.1
*D.castaneae*	10.4–12.3	2.2–2.7	4.2–5.2
*D.castaneicola*	10.5–12.8	3.2–3.8	3–4
*D.chinense*	7.7–9.1	3.4–3.7	2.2–2.6
*D.parasiticum*	9.3–11.7	2.8–3.3	3–3.9
*D.rizhaoense*	6.4–8.8	2.7–3.8	1.8–3.1
*D.shaanxiense*	9.5–11.1	2.5–3.1	3.3–4.2
*D.shandongense*	8.1–8.8	3.8–4.3	1.9–2.3
*D.tianii*	9.5–11.1	2.6–3.2	3.2–4.1

Morphological identification for *Dendrostoma* becomes difficult and host and geographical data are obvious unuseful because most species are host-overlapped. Besides, most species are only known in asexual morph. Hence, DNA sequence data are necessary during species identification. LSU is proposed as the genus DNA barcode, and ITS, *tef1* and *rpb2* as the species DNA barcode (Chen et al. 2022).

*Dendrostoma* is a young diaporthalean genus established recently, with typical characters of Diaporthales (Castlebury et al. 2002; Senanayake et al. 2017; Fan et al. 2018; Jiang et al. 2020). For example, *D.atlanticum* and *D.quercus* have dimorphic conidia like *Diaporthe* (Yang et al. 2018, 2021); most species of this genus have central column beneath the conidiomata like *Melanconis* (Voglmayr et al. 2012; Jiang et al. 2018, 2021). Hence, *D.leiphaemia* was classified in *Amphiporthe* and *D.castaneum* in *Valsa* (Senanayake et al. 2018; Jaklitsch and Voglmayr 2019). Recent studies of *Dendrostoma* largely improved the understandings of genus and family concepts in Diaporthales.

## Supplementary Material

XML Treatment for Dendrostoma rizhaoense

XML Treatment for Dendrostoma tianii

## ﻿Additional information

### Author ORCIDs

Ning Jiang https://orcid.org/0000-0002-9656-8500

Han Xue https://orcid.org/0000-0003-0414-6237

Yong Li https://orcid.org/0000-0002-4406-1329

### Data availability

All of the data that support the findings of this study are available in the main text.
